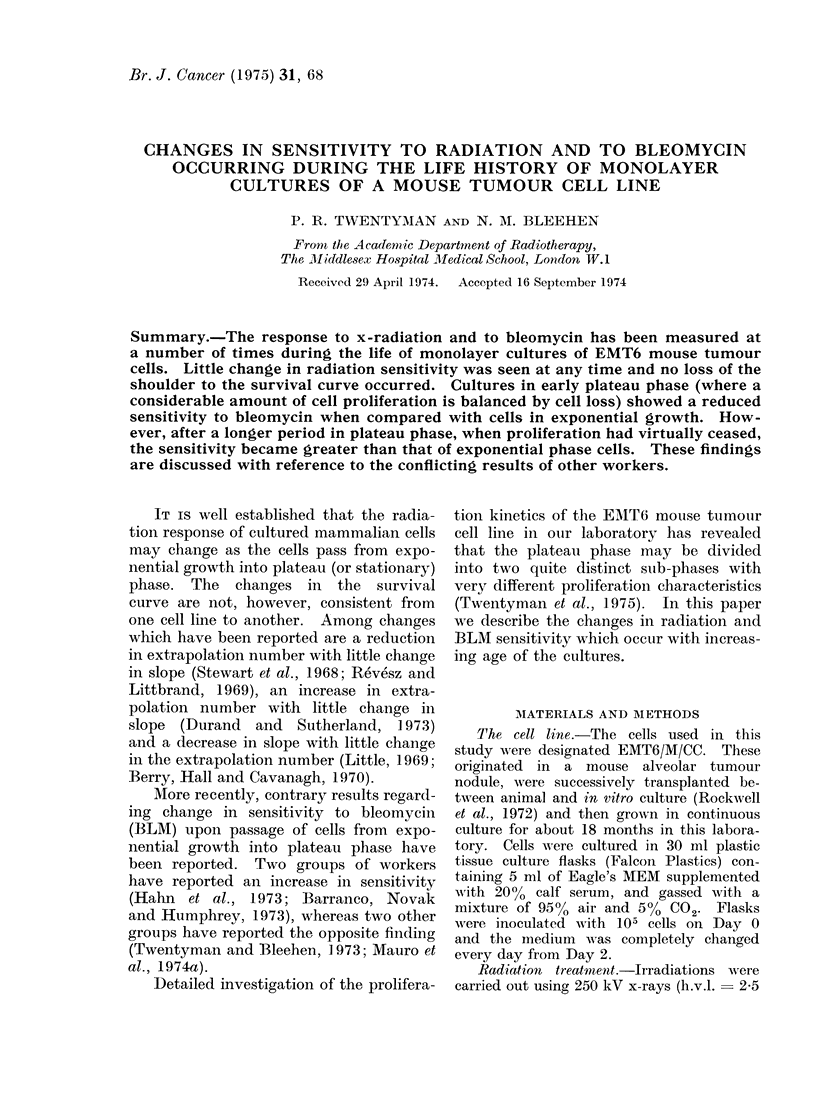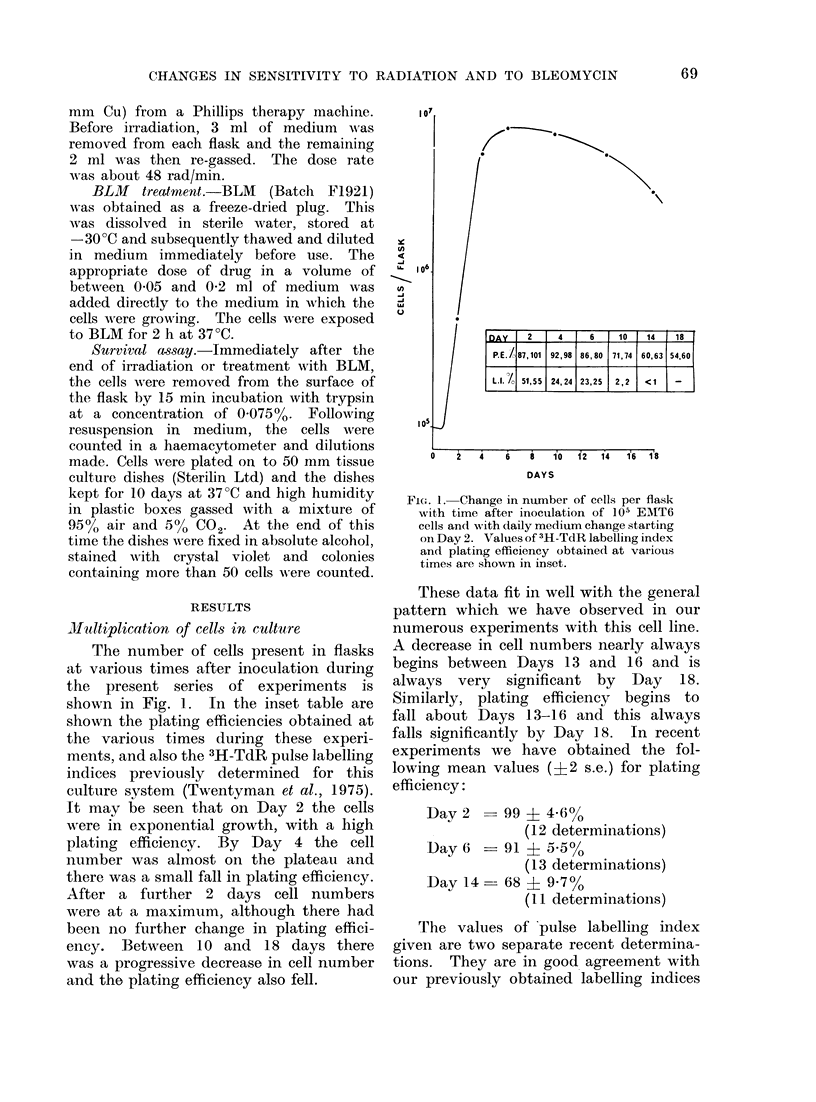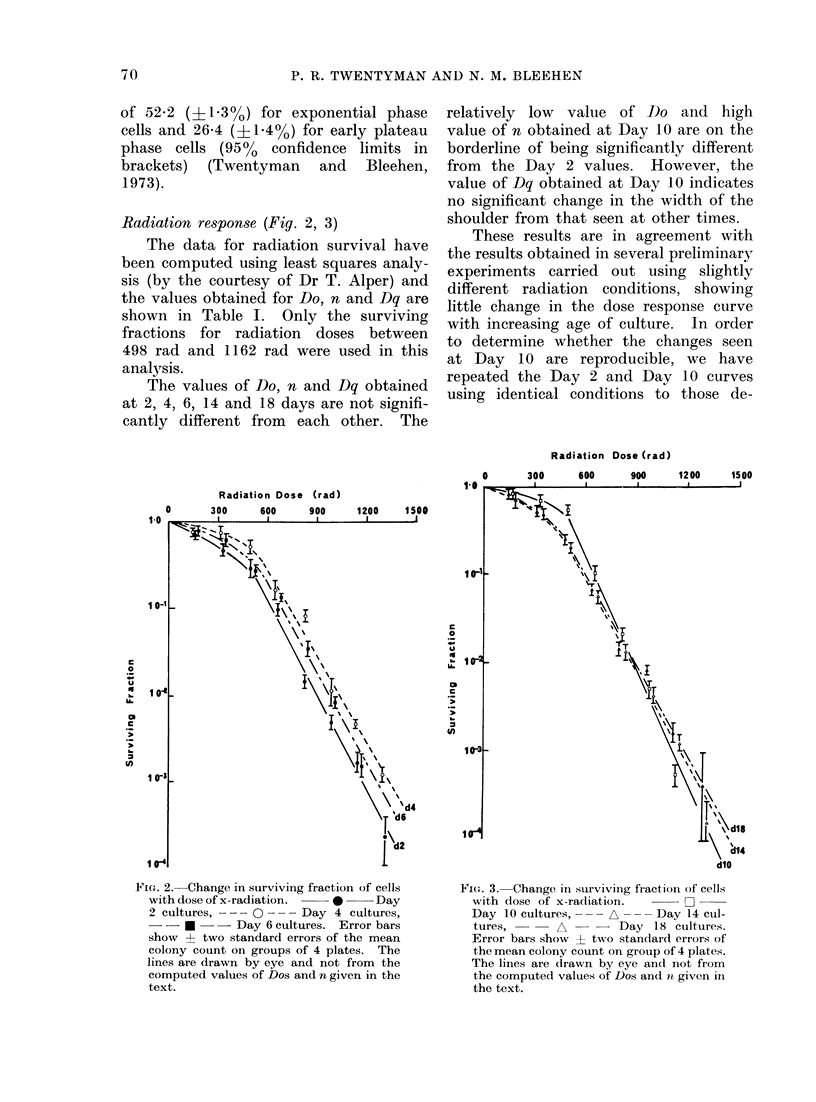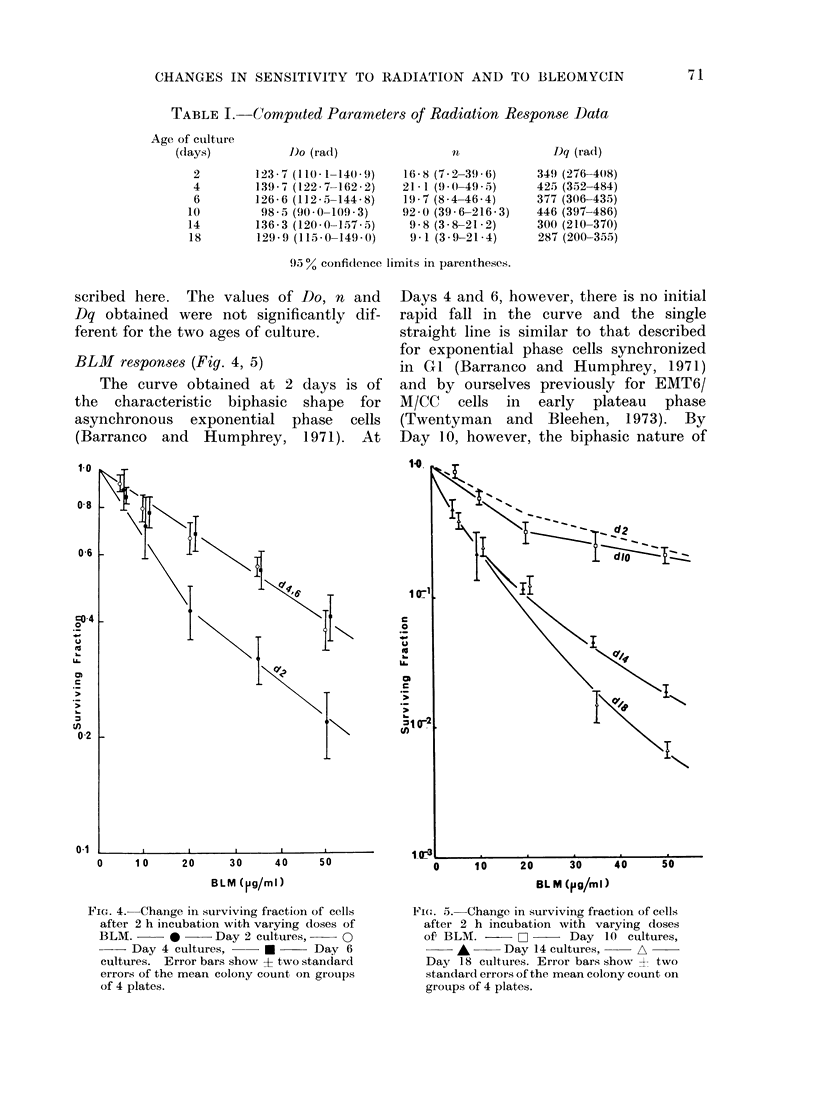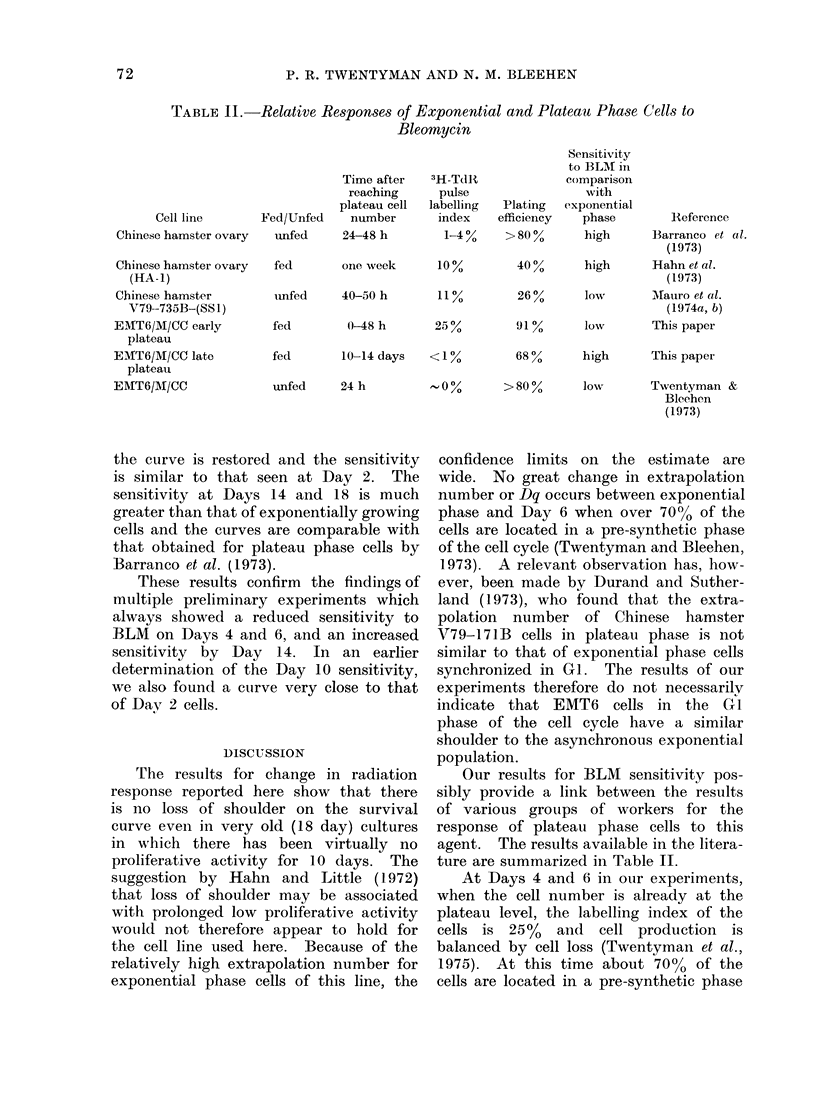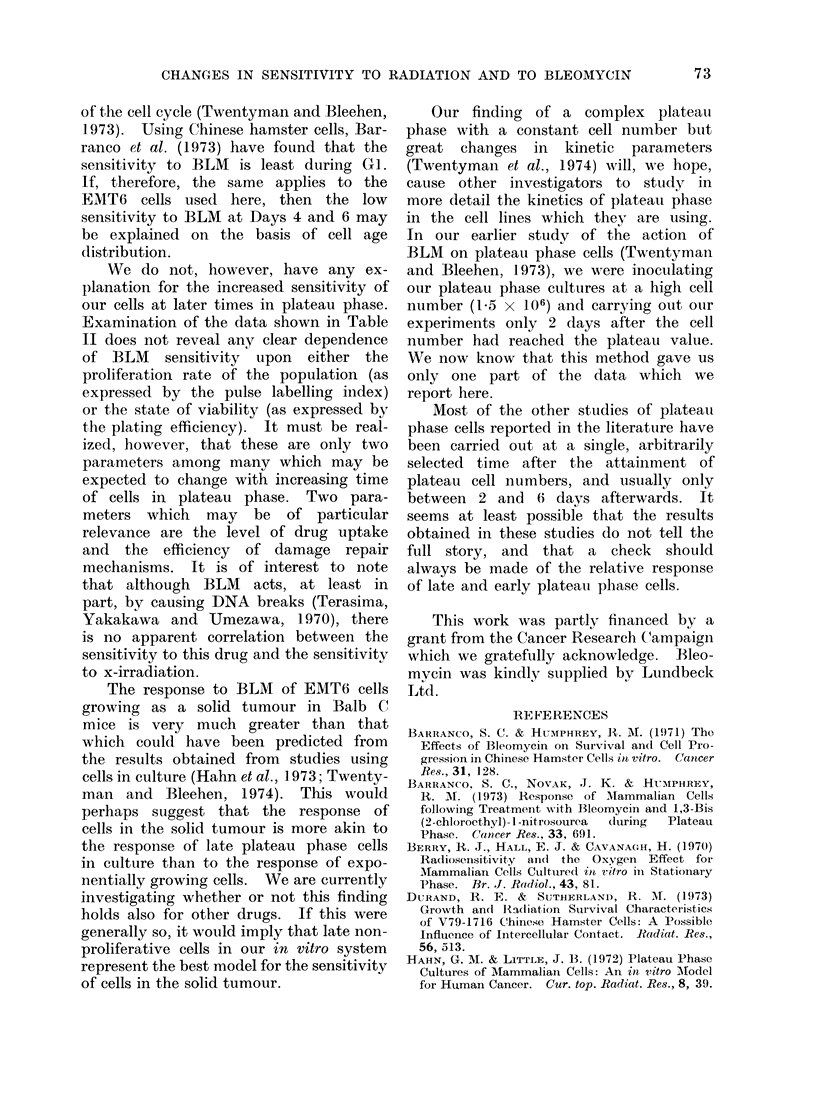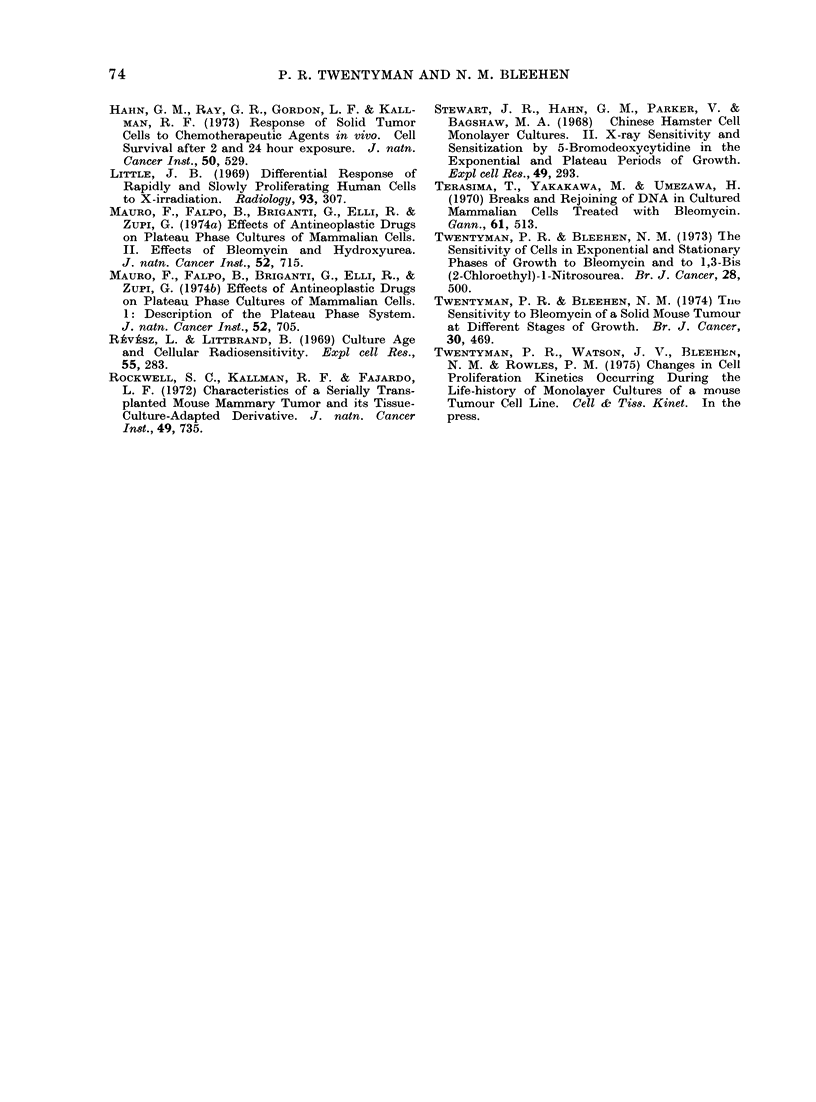# Changes in sensitivity to radiation and to blemycin occurring during the life history of monolayer cultures of a mouse tumour cell line.

**DOI:** 10.1038/bjc.1975.8

**Published:** 1975-01

**Authors:** P. R. Twentyman, N. M. Bleehen

## Abstract

The response to X-radiation and to bleomycin has been measured at a number of times during the life of monolayer cultures of EMT6 mouse tumour cells. Little change in radiation sensitivity was seen at any time and no loss of the shoulder to the survival curve occurred. Cultures in early plateau phase (where a considerable amount of cell proliferation is balanced by cell loss) showed a reduced sensitivity to bleomycin when compared with cells in exponential growth. However, after a longer period in plateau phase, when proliferation had virtually ceased, the sensitivity became greater than that of exponetial phase cells. These findings are discussed wirh reference to the conflicting results of other workers.


*Br. J. Cancer* (1975) **31**, 68

### CHANGES IN SENSITIVITY TO RADIATION AND TO BLEOMYCIN OCCURRING DURING THE LIFE HISTORY OF MONOLAYER CULTURES OF A MOUSE TUMOUR CELL LINE

P. R. TWENTYMAN AND N. M. BLEEHEN

*From the Academic Department of Radiotherapy, The Middlesex Hospital Medical School, London W.1*

Received 29 April 1974. Accepted 16 September 1974

**Summary.—The response to x-radiation and to bleomycin has been measured at a number of times during the life of monolayer cultures of EMT6 mouse tumour cells. Little change in radiation sensitivity was seen at any time and no loss of the shoulder to the survival curve occurred. Cultures in early plateau phase (where a considerable amount of cell proliferation is balanced by cell loss) showed a reduced sensitivity to bleomycin when compared with cells in exponential growth. However, after a longer period in plateau phase, when proliferation had virtually ceased, the sensitivity became greater than that of exponential phase cells. These findings are discussed with reference to the conflicting results of other workers.**

IT IS well established that the radiation response of cultured mammalian cells may change as the cells pass from exponential growth into plateau (or stationary) phase. The changes in the survival curve are not, however, consistent from one cell line to another. Among changes which have been reported are a reduction in extrapolation number with little change in slope (Stewart *et al.*, 1968; Révész and Littbrand, 1969), an increase in extrapolation number with little change in slope (Durand and Sutherland, 1973) and a decrease in slope with little change in the extrapolation number (Little, 1969; Berry, Hall and Cavanagh, 1970).

More recently, contrary results regarding change in sensitivity to bleomycin (BLM) upon passage of cells from exponential growth into plateau phase have been reported. Two groups of workers have reported an increase in sensitivity (Hahn *et al.*, 1973; Barranco, Novak and Humphrey, 1973), whereas two other groups have reported the opposite finding (Twentyman and Bleehen, 1973; Mauro *et al.*, 1974*a*).

Detailed investigation of the proliferation kinetics of the EMT6 mouse tumour cell line in our laboratory has revealed that the plateau phase may be divided into two quite distinct sub-phases with very different proliferation characteristics (Twentyman *et al.*, 1975). In this paper we describe the changes in radiation and BLM sensitivity which occur with increasing age of the cultures.

### MATERIALS AND METHODS

*The cell line.*—The cells used in this study were designated EMT6/M/CC. These originated in a mouse alveolar tumour nodule, were successively transplanted between animal and *in vitro* culture (Rockwell *et al.*, 1972) and then grown in continuous culture for about 18 months in this laboratory. Cells were cultured in 30 ml plastic tissue culture flasks (Falcon Plastics) containing 5 ml of Eagle's MEM supplemented with 20% calf serum, and gassed with a mixture of 95% air and 5% $CO_2$. Flasks were inoculated with $10^5$ cells on Day 0 and the medium was completely changed every day from Day 2.

*Radiation treatment.*—Irradiations were carried out using 250 kV x-rays (h.v.l. = 2·5

mm Cu) from a Phillips therapy machine. Before irradiation, 3 ml of medium was removed from each flask and the remaining 2 ml was then re-gassed. The dose rate was about 48 rad/min.

*BLM treatment.*—BLM (Batch F1921) was obtained as a freeze-dried plug. This was dissolved in sterile water, stored at −30 °C and subsequently thawed and diluted in medium immediately before use. The appropriate dose of drug in a volume of between 0·05 and 0·2 ml of medium was added directly to the medium in which the cells were growing. The cells were exposed to BLM for 2 h at 37 °C.

*Survival assay.*—Immediately after the end of irradiation or treatment with BLM, the cells were removed from the surface of the flask by 15 min incubation with trypsin at a concentration of 0·075%. Following resuspension in medium, the cells were counted in a haemacytometer and dilutions made. Cells were plated on to 50 mm tissue culture dishes (Sterilin Ltd) and the dishes kept for 10 days at 37 °C and high humidity in plastic boxes gassed with a mixture of 95% air and 5% $CO_2$. At the end of this time the dishes were fixed in absolute alcohol, stained with crystal violet and colonies containing more than 50 cells were counted.

| DAY | 2 | 4 | 6 | 10 | 14 | 18 |
|---|---|---|---|---|---|---|
| P.E.% | 87,101 | 92,98 | 86,80 | 71,74 | 60,63 | 54,60 |
| L.I.% | 51,55 | 24,24 | 23,25 | 2,2 | <1 | – |

FIG. 1.—Change in number of cells per flask with time after inoculation of $10^5$ EMT6 cells and with daily medium change starting on Day 2. Values of $^3$H-TdR labelling index and plating efficiency obtained at various times are shown in inset.

### RESULTS

### *Multiplication of cells in culture*

The number of cells present in flasks at various times after inoculation during the present series of experiments is shown in Fig. 1. In the inset table are shown the plating efficiencies obtained at the various times during these experiments, and also the $^3$H-TdR pulse labelling indices previously determined for this culture system (Twentyman *et al.*, 1975). It may be seen that on Day 2 the cells were in exponential growth, with a high plating efficiency. By Day 4 the cell number was almost on the plateau and there was a small fall in plating efficiency. After a further 2 days cell numbers were at a maximum, although there had been no further change in plating efficiency. Between 10 and 18 days there was a progressive decrease in cell number and the plating efficiency also fell.

These data fit in well with the general pattern which we have observed in our numerous experiments with this cell line. A decrease in cell numbers nearly always begins between Days 13 and 16 and is always very significant by Day 18. Similarly, plating efficiency begins to fall about Days 13–16 and this always falls significantly by Day 18. In recent experiments we have obtained the following mean values (±2 s.e.) for plating efficiency:

Day 2 = 99 ± 4·6%

(12 determinations)

Day 6 = 91 ± 5·5%

(13 determinations)

Day 14 = 68 ± 9·7%

(11 determinations)

The values of pulse labelling index given are two separate recent determinations. They are in good agreement with our previously obtained labelling indices of 52·2 ($\pm$1·3%) for exponential phase cells and 26·4 ($\pm$1·4%) for early plateau phase cells (95% confidence limits in brackets) (Twentyman and Bleehen, 1973).

*Radiation response* (*Fig.* 2, 3)

The data for radiation survival have been computed using least squares analysis (by the courtesy of Dr T. Alper) and the values obtained for *Do*, *n* and *Dq* are shown in Table I. Only the surviving fractions for radiation doses between 498 rad and 1162 rad were used in this analysis.

The values of *Do*, *n* and *Dq* obtained at 2, 4, 6, 14 and 18 days are not significantly different from each other. The relatively low value of *Do* and high value of *n* obtained at Day 10 are on the borderline of being significantly different from the Day 2 values. However, the value of *Dq* obtained at Day 10 indicates no significant change in the width of the shoulder from that seen at other times.

These results are in agreement with the results obtained in several preliminary experiments carried out using slightly different radiation conditions, showing little change in the dose response curve with increasing age of culture. In order to determine whether the changes seen at Day 10 are reproducible, we have repeated the Day 2 and Day 10 curves using identical conditions to those de-

FIG. 2.—Change in surviving fraction of cells with dose of x-radiation. —— ● —— Day 2 cultures, - - - ○ - - - Day 4 cultures, — — ■ — — Day 6 cultures. Error bars show $\pm$ two standard errors of the mean colony count on groups of 4 plates. The lines are drawn by eye and not from the computed values of *Do*s and *n* given in the text.

FIG. 3.—Change in surviving fraction of cells with dose of x-radiation. —— □ —— Day 10 cultures, - - - △ - - - Day 14 cultures, — — △ — — Day 18 cultures. Error bars show $\pm$ two standard errors of the mean colony count on group of 4 plates. The lines are drawn by eye and not from the computed values of *Do*s and *n* given in the text.

TABLE I.—*Computed Parameters of Radiation Response Data*

| Age of culture (days) | *Do* (rad) | *n* | *Dq* (rad) |
|---|---|---|---|
| 2 | 123·7 (110·1–140·9) | 16·8 (7·2–39·6) | 349 (276–408) |
| 4 | 139·7 (122·7–162·2) | 21·1 (9·0–49·5) | 425 (352–484) |
| 6 | 126·6 (112·5–144·8) | 19·7 (8·4–46·4) | 377 (306–435) |
| 10 | 98·5 (90·0–109·3) | 92·0 (39·6–216·3) | 446 (397–486) |
| 14 | 136·3 (120·0–157·5) | 9·8 (3·8–21·2) | 300 (210–370) |
| 18 | 129·9 (115·0–149·0) | 9·1 (3·9–21·4) | 287 (200–355) |

95% confidence limits in parentheses.

scribed here. The values of *Do*, *n* and *Dq* obtained were not significantly different for the two ages of culture.

*BLM responses* (*Fig.* 4, 5)

The curve obtained at 2 days is of the characteristic biphasic shape for asynchronous exponential phase cells (Barranco and Humphrey, 1971). At Days 4 and 6, however, there is no initial rapid fall in the curve and the single straight line is similar to that described for exponential phase cells synchronized in G1 (Barranco and Humphrey, 1971) and by ourselves previously for EMT6/M/CC cells in early plateau phase (Twentyman and Bleehen, 1973). By Day 10, however, the biphasic nature of

FIG. 4.—Change in surviving fraction of cells after 2 h incubation with varying doses of BLM. —— ● —— Day 2 cultures, —— ○ —— Day 4 cultures, —— ■ —— Day 6 cultures. Error bars show ± two standard errors of the mean colony count on groups of 4 plates.

FIG. 5.—Change in surviving fraction of cells after 2 h incubation with varying doses of BLM. —— □ —— Day 10 cultures, —— ▲ —— Day 14 cultures, —— △ —— Day 18 cultures. Error bars show ± two standard errors of the mean colony count on groups of 4 plates.

TABLE II.—*Relative Responses of Exponential and Plateau Phase Cells to Bleomycin*

| Cell line | Fed/Unfed | Time after reaching plateau cell number | $^3$H-TdR pulse labelling index | Plating efficiency | Sensitivity to BLM in comparison with exponential phase | Reference |
|---|---|---|---|---|---|---|
| Chinese hamster ovary | unfed | 24–48 h | 1–4% | >80% | high | Barranco *et al.* (1973) |
| Chinese hamster ovary (HA-1) | fed | one week | 10% | 40% | high | Hahn *et al.* (1973) |
| Chinese hamster V79–735B–(SS1) | unfed | 40–50 h | 11% | 26% | low | Mauro *et al.* (1974*a*, *b*) |
| EMT6/M/CC early plateau | fed | 0–48 h | 25% | 91% | low | This paper |
| EMT6/M/CC late plateau | fed | 10–14 days | <1% | 68% | high | This paper |
| EMT6/M/CC | unfed | 24 h | ~0% | >80% | low | Twentyman & Bleehen (1973) |

the curve is restored and the sensitivity is similar to that seen at Day 2. The sensitivity at Days 14 and 18 is much greater than that of exponentially growing cells and the curves are comparable with that obtained for plateau phase cells by Barranco *et al.* (1973).

These results confirm the findings of multiple preliminary experiments which always showed a reduced sensitivity to BLM on Days 4 and 6, and an increased sensitivity by Day 14. In an earlier determination of the Day 10 sensitivity, we also found a curve very close to that of Day 2 cells.

### DISCUSSION

The results for change in radiation response reported here show that there is no loss of shoulder on the survival curve even in very old (18 day) cultures in which there has been virtually no proliferative activity for 10 days. The suggestion by Hahn and Little (1972) that loss of shoulder may be associated with prolonged low proliferative activity would not therefore appear to hold for the cell line used here. Because of the relatively high extrapolation number for exponential phase cells of this line, the confidence limits on the estimate are wide. No great change in extrapolation number or *Dq* occurs between exponential phase and Day 6 when over 70% of the cells are located in a pre-synthetic phase of the cell cycle (Twentyman and Bleehen, 1973). A relevant observation has, however, been made by Durand and Sutherland (1973), who found that the extrapolation number of Chinese hamster V79–171B cells in plateau phase is not similar to that of exponential phase cells synchronized in G1. The results of our experiments therefore do not necessarily indicate that EMT6 cells in the G1 phase of the cell cycle have a similar shoulder to the asynchronous exponential population.

Our results for BLM sensitivity possibly provide a link between the results of various groups of workers for the response of plateau phase cells to this agent. The results available in the literature are summarized in Table II.

At Days 4 and 6 in our experiments, when the cell number is already at the plateau level, the labelling index of the cells is 25% and cell production is balanced by cell loss (Twentyman *et al.*, 1975). At this time about 70% of the cells are located in a pre-synthetic phase of the cell cycle (Twentyman and Bleehen, 1973). Using Chinese hamster cells, Barranco *et al.* (1973) have found that the sensitivity to BLM is least during G1. If, therefore, the same applies to the EMT6 cells used here, then the low sensitivity to BLM at Days 4 and 6 may be explained on the basis of cell age distribution.

We do not, however, have any explanation for the increased sensitivity of our cells at later times in plateau phase. Examination of the data shown in Table II does not reveal any clear dependence of BLM sensitivity upon either the proliferation rate of the population (as expressed by the pulse labelling index) or the state of viability (as expressed by the plating efficiency). It must be realized, however, that these are only two parameters among many which may be expected to change with increasing time of cells in plateau phase. Two parameters which may be of particular relevance are the level of drug uptake and the efficiency of damage repair mechanisms. It is of interest to note that although BLM acts, at least in part, by causing DNA breaks (Terasima, Yakakawa and Umezawa, 1970), there is no apparent correlation between the sensitivity to this drug and the sensitivity to x-irradiation.

The response to BLM of EMT6 cells growing as a solid tumour in Balb C mice is very much greater than that which could have been predicted from the results obtained from studies using cells in culture (Hahn *et al.*, 1973; Twentyman and Bleehen, 1974). This would perhaps suggest that the response of cells in the solid tumour is more akin to the response of late plateau phase cells in culture than to the response of exponentially growing cells. We are currently investigating whether or not this finding holds also for other drugs. If this were generally so, it would imply that late non-proliferative cells in our *in vitro* system represent the best model for the sensitivity of cells in the solid tumour.

Our finding of a complex plateau phase with a constant cell number but great changes in kinetic parameters (Twentyman *et al.*, 1974) will, we hope, cause other investigators to study in more detail the kinetics of plateau phase in the cell lines which they are using. In our earlier study of the action of BLM on plateau phase cells (Twentyman and Bleehen, 1973), we were inoculating our plateau phase cultures at a high cell number ($1{\cdot}5 \times 10^6$) and carrying out our experiments only 2 days after the cell number had reached the plateau value. We now know that this method gave us only one part of the data which we report here.

Most of the other studies of plateau phase cells reported in the literature have been carried out at a single, arbitrarily selected time after the attainment of plateau cell numbers, and usually only between 2 and 6 days afterwards. It seems at least possible that the results obtained in these studies do not tell the full story, and that a check should always be made of the relative response of late and early plateau phase cells.

This work was partly financed by a grant from the Cancer Research Campaign which we gratefully acknowledge. Bleomycin was kindly supplied by Lundbeck Ltd.

REFERENCES

Barranco, S. C. & Humphrey, R. M. (1971) The Effects of Bleomycin on Survival and Cell Progression in Chinese Hamster Cells *in vitro*. *Cancer Res.*, **31**, 128.

Barranco, S. C., Novak, J. K. & Humphrey, R. M. (1973) Response of Mammalian Cells following Treatment with Bleomycin and 1,3-Bis (2-chloroethyl)-1-nitrosourea during Plateau Phase. *Cancer Res.*, **33**, 691.

Berry, R. J., Hall, E. J. & Cavanagh, H. (1970) Radiosensitivity and the Oxygen Effect for Mammalian Cells Cultured *in vitro* in Stationary Phase. *Br. J. Radiol.*, **43**, 81.

Durand, R. E. & Sutherland, R. M. (1973) Growth and Radiation Survival Characteristics of V79-1716 Chinese Hamster Cells: A Possible Influence of Intercellular Contact. *Radiat. Res.*, **56**, 513.

Hahn, G. M. & Little, J. B. (1972) Plateau Phase Cultures of Mammalian Cells: An *in vitro* Model for Human Cancer. *Cur. top. Radiat. Res.*, **8**, 39.

HAHN, G. M., RAY, G. R., GORDON, L. F. & KALLMAN, R. F. (1973) Response of Solid Tumor Cells to Chemotherapeutic Agents *in vivo*. Cell Survival after 2 and 24 hour exposure. *J. natn. Cancer Inst.*, **50**, 529.

LITTLE, J. B. (1969) Differential Response of Rapidly and Slowly Proliferating Human Cells to X-irradiation. *Radiology*, **93**, 307.

MAURO, F., FALPO, B., BRIGANTI, G., ELLI, R. & ZUPI, G. (1974*a*) Effects of Antineoplastic Drugs on Plateau Phase Cultures of Mammalian Cells. II. Effects of Bleomycin and Hydroxyurea. *J. natn. Cancer Inst.*, **52**, 715.

MAURO, F., FALPO, B., BRIGANTI, G., ELLI, R., & ZUPI, G. (1974*b*) Effects of Antineoplastic Drugs on Plateau Phase Cultures of Mammalian Cells. 1: Description of the Plateau Phase System. *J. natn. Cancer Inst.*, **52**, 705.

RÉVÉSZ, L. & LITTBRAND, B. (1969) Culture Age and Cellular Radiosensitivity. *Expl cell Res.*, **55**, 283.

ROCKWELL, S. C., KALLMAN, R. F. & FAJARDO, L. F. (1972) Characteristics of a Serially Transplanted Mouse Mammary Tumor and its Tissue-Culture-Adapted Derivative. *J. natn. Cancer Inst.*, **49**, 735.

STEWART, J. R., HAHN, G. M., PARKER, V. & BAGSHAW, M. A. (1968) Chinese Hamster Cell Monolayer Cultures. II. X-ray Sensitivity and Sensitization by 5-Bromodeoxycytidine in the Exponential and Plateau Periods of Growth. *Expl cell Res.*, **49**, 293.

TERASIMA, T., YAKAKAWA, M. & UMEZAWA, H. (1970) Breaks and Rejoining of DNA in Cultured Mammalian Cells Treated with Bleomycin. *Gann.*, **61**, 513.

TWENTYMAN, P. R. & BLEEHEN, N. M. (1973) The Sensitivity of Cells in Exponential and Stationary Phases of Growth to Bleomycin and to 1,3-Bis (2-Chloroethyl)-1-Nitrosourea. *Br. J. Cancer*, **28**, 500.

TWENTYMAN, P. R. & BLEEHEN, N. M. (1974) The Sensitivity to Bleomycin of a Solid Mouse Tumour at Different Stages of Growth. *Br. J. Cancer*, **30**, 469.

TWENTYMAN, P. R., WATSON, J. V., BLEEHEN, N. M. & ROWLES, P. M. (1975) Changes in Cell Proliferation Kinetics Occurring During the Life-history of Monolayer Cultures of a mouse Tumour Cell Line. *Cell & Tiss. Kinet.* In the press.